# Telemedicine Booths for Screening Cardiovascular Risk Factors: Prospective Multicenter Study

**DOI:** 10.2196/57032

**Published:** 2025-04-22

**Authors:** Mélanie Decambron, Christine Tchikladze Merand

**Affiliations:** 1 Polyclinique Vauban, Elsan, Valenciennes, France; 2 Elsan, 58 bis rue de la Boétie, Paris, 75008, France, 33 785887789

**Keywords:** cardiovascular disease risk factors, cardiovascular disease, CVD, cardiology, cardiac, cardiologists, health check, hypertension, HTN, hypertensive, high blood pressure, blood pressure, obesity, screening, health screening, telemedicine, telehealth, virtual care, virtual health, virtual medicine, COVID-19, SARS-CoV-2, Coronavirus, respiratory, infectious, pulmonary, pandemic

## Abstract

**Background:**

Cardiovascular risk factors such as hypertension often remain undetected and untreated. This was particularly problematic during the COVID-19 pandemic when there were fewer in-person medical consultations.

**Objective:**

This study aimed to determine whether health screening using a telemedicine booth would have an impact on people’s medical care during the COVID-19 pandemic.

**Methods:**

Health screening was run using a telemedicine booth (the consult station) that was placed in three different vaccination centers in northern France between July 2021 and September 2021. Participants followed a series of instructions to obtain various measures, including their blood pressure, BMI, and heart rate. If any measures were found to be outside of the normal range, participants were advised to consult a doctor. After 3 months, the participants with abnormal readings were contacted by telephone and were asked a series of standardized questions. The primary outcome was the percentage of respondents who reported that they had consulted a doctor since the health check.

**Results:**

Approximately 6000 people attended the 3 vaccination centers over the study period. Of these, around 2500 used the consult station. A total of 1333 participants (53.3%) were found to have abnormal readings, which mostly concerned their blood pressure, heart rate, or BMI. There were 638 participants who responded to the follow-up call, and 234 of these (37%) reported that they had consulted a doctor since the health check. However, 158 of the 638 respondents (24.8%) reported that they would have consulted a doctor even without the screening.

**Conclusions:**

We succeeded in screening large numbers of people for cardiovascular risk factors during the COVID-19 pandemic by using a telemedicine booth. Although relatively few follow-up call respondents reported that they went on to consult a physician, the screening would nevertheless have raised people’s awareness of their cardiovascular risk factors.

## Introduction

Cardiovascular disease (CVD) is the leading cause of death worldwide [1]. In France, the disease affects around 7.9% of the population and causes around 140,000 deaths each year, which is only exceeded by deaths caused by cancer [2,3]. The health care costs are substantial, representing around 10% of the country’s health care reimbursements [4]. The burden of CVD is likely to increase over the coming years due to an aging population and is thus a major cause for concern [4].

There are several well-known modifiable risk factors for CVD, including obesity, high blood pressure, and physical inactivity [5]. The most significant of these is hypertension because it accounts for the largest number of CVD deaths [5,6]. In France, this affects around 22% of the population, but over a third are unaware that they have the condition because there are usually no symptoms [4,7]. As a result, many people do not benefit from interventions, such as antihypertensive medication, that can effectively lower their blood pressure, thus reducing the risk of CVD [8]. Obesity is another important modifiable risk factor for CVD, which affects 21.6% of the adult population in France [4]. Although effective treatment options are available, such as lifestyle modifications, antiobesity medication, and bariatric surgery, people with obesity typically believe that it is their responsibility to lose weight and so often wait many years before seeking medical help [9-11].

Screening for CVD risk factors is of much importance for public health, as it enables risk factors such as hypertension to be detected and treated, which can prevent CVD. Most screening takes place during medical consultations with a general practitioner (GP), where blood pressure and weight are regularly monitored. However, some people rarely consult a medical professional. For instance, a study of over 8000 adults in England found that 22.8% (1846/8086) had not consulted a GP over the last year [12]. This means that CVD risk factors may remain undetected and untreated. In addition, the lack of GPs in certain regions is likely to further limit the number of people who are screened [13,14].

Various screening programs have been set up over the years to target people who rarely consult a GP. These programs have often focused on detecting hypertension, also known as the “silent killer,” and they have been run in a variety of different settings [15-17]. Some screening programs have involved health checks carried out by nurses, pharmacists, or medical students in settings such as pharmacies, dental surgeries, and community centers [18]. However, these programs may not always be feasible, such as during pandemics when strict social distancing requirements are in place. In addition, they are likely to be limited by funding and staffing resources. As an alternative solution, self-screening facilities have been developed, which have the advantage of lower running costs as well as the potential to be used during pandemics [19]. For instance, health kiosks that assess blood pressure and weight are now available in pharmacies and shopping centers throughout the United States, including more than 6000 health stations developed by the company Higi (Chicago, United States). There is evidence that these have a positive impact. For instance, a study showed that 27.4% (17/62) of users with hypertension achieved blood pressure control within 6 to 12 months [20]. These rates could potentially be improved through patient education and by referring people on to treatment programs, such as the Kaiser Permanente treatment protocol [20,21]. However, health kiosks are not widely available in many countries, including France. Other possibilities for screening include the use of personal digital devices, such as smartphones; however, not all people have these and there are concerns about their accuracy [22].

Recently, a telemedicine booth was developed in France (the consult station, H4D, Paris) that can run a series of health checks in the privacy of a stand-alone booth [23]. The tests can be run by users without any input from a medical professional; there is also the option of using the device for teleconsultations with a physician. The booth can be used to assess blood pressure, BMI, and heart rate, which have all been linked to CVD [5,24]. In addition, other measures can also be obtained that can signal other health conditions, such as oxygen saturation, where low levels could point to anemia or pulmonary diseases, and rapid visual tests, where abnormalities could be a sign of macular degeneration or epiretinal membrane.

In this study, we assessed the feasibility and interest of using the consult station telemedicine booth for self-screening during the COVID-19 pandemic, when there was a particular need for remote medical services. The booths were placed in several vaccination centers throughout northern France at a time when large numbers of people were coming to be vaccinated. Our primary aim was to determine whether screening using the telemedicine booth would have an impact on the medical care of people with CVD risk factors. We also aimed to determine whether people who were not registered with a GP (11% of people in France) [13] had more CVD risk factors and whether they would be more likely to consult a medical professional following the screening.

## Methods

### Study Design

This study was a prospective, single-arm, multicenter study. All participants provided informed consent before taking part in the study; this concerned all those who had a health check using the consult station. Participants were also sent an information sheet by post or email before the follow-up telephone interview, which included a form to complete and return if they did not agree to their data being used for the study.

### Setting

This study was carried out in 3 different vaccination centers between July 1, 2021, and July 1, 2022. The centers were all located in hospitals in northern France.

### Participants

Participants were included in the study if they were aged 18 years or older and were affiliated to a social security scheme. Participants were excluded if they could not follow the instructions in the consult station or if they were under guardianship. There was no sample size calculation for this study.

### Screening

During the waiting periods at the vaccination centers (before or after vaccination, or at any time for accompanying adults), people were offered a free health check using the consult station, a telemedicine booth developed by H4D (Paris, France). This is a class IIa medical device that can measure people’s blood pressure, heart rate, oxygen saturation, BMI, and vision (acuity and central visual field using the Amsler grid) in less than 15 minutes. The health check is run by following a series of instructions on a screen, and it does not require the involvement of medical staff. However, a nurse was present to interpret the results, which were provided on a printed sheet of paper, and participants were advised to see a doctor if there were any readings outside of the normal range. For the purposes of this study, the following readings were considered to be outside the normal range: BMI >30 kg/m^2^ (obese: 31‐40 kg/m^2^; severely obese >40 kg/m^2^), heart rate >80 beats per minute (bpm) or <50 bpm (high heart rate: 81‐100 bpm; tachycardia: >100bpm), oxygen saturation <94% (mild: 90‐93%; hypoxia: <90%), systolic blood pressure <100mmHg or >140mmHg (high: 141‐180mmHg; very high: >180mmHg), and diastolic blood pressure <70 mmHg or >90 mmHg (high: 91‐100mmHg; very high: >100mmHg). The Amsler grid results were marked as abnormal if the participants saw the grid to be distorted (eg, wavy lines) when focusing on the central dot. We took the contact details (eg, telephone and email) of the participants who had screening results outside the normal range, with their permission. These participants were then contacted 3 months later to evaluate the impact of the screening.

### Follow-Up

Three months after the health check, the participants were contacted by telephone and were asked to respond to a series of questions. These covered their demographic details (sex and history of smoking), their medical history before the health check (hypertension, cardiac arrhythmia, pulmonary disease, diabetes, or sleep apnea; whether they took medication), their reason for coming to the vaccination center (to be vaccinated, accompanying someone, or member of staff), whether they were registered with a GP, and how often they saw a medical professional. Participants were also asked whether they had consulted a doctor since the health check, and if so, whether this was with their GP. They were also asked whether the doctor had confirmed the cardiovascular risk factors and initiated regular follow-up appointments to monitor these. They were also asked whether they would have consulted a doctor without the health check (yes or no). Additional questions covered whether they had seen or planned to see a medical specialist, or whether they had been told that this was not necessary. Participants were also asked whether they had been to see a dietician. If they were smokers, they were also asked whether they had stopped smoking or taken steps to do so. Participants were also asked whether their health check had been before or after vaccination (or during vaccination for those accompanying others).

### Outcome Measures

The primary outcome was the percentage of participants with abnormal screening results who then consulted a doctor (GP or specialist). The secondary outcome was the percentage of participants not registered with a GP who then consulted a doctor (GP or specialist) following abnormal screening results. An additional secondary outcome was the difference in cardiovascular risk factors between participants who were registered with a GP and those who were not registered with a GP.

### Data Analysis

The program R (R Foundation for Statistical Computing) was used for the statistical analyses. The qualitative variables were summarized using frequencies and percentages; quantitative variables were summarized using the means and standard deviations, or medians, inter-quartile ranges (IQR), and ranges. Statistical analyses were run according to the type of data (eg, *χ*^2^ or Fisher exact tests for categorical data) using a significance level of 0.05. We also ran univariate and multivariate analyses to identify factors that could predict whether the participants would consult a doctor following screening.

### Ethical Considerations

The study was approved by the ELSAN Institutional Review Board (#2022-03-Dr DECAMBRON-01; approved on March 21, 2022) and is registered on the Health Data Hub (number: F20220309155706. All participants provided informed consent.

## Results

### Participants

**Figure 1.**
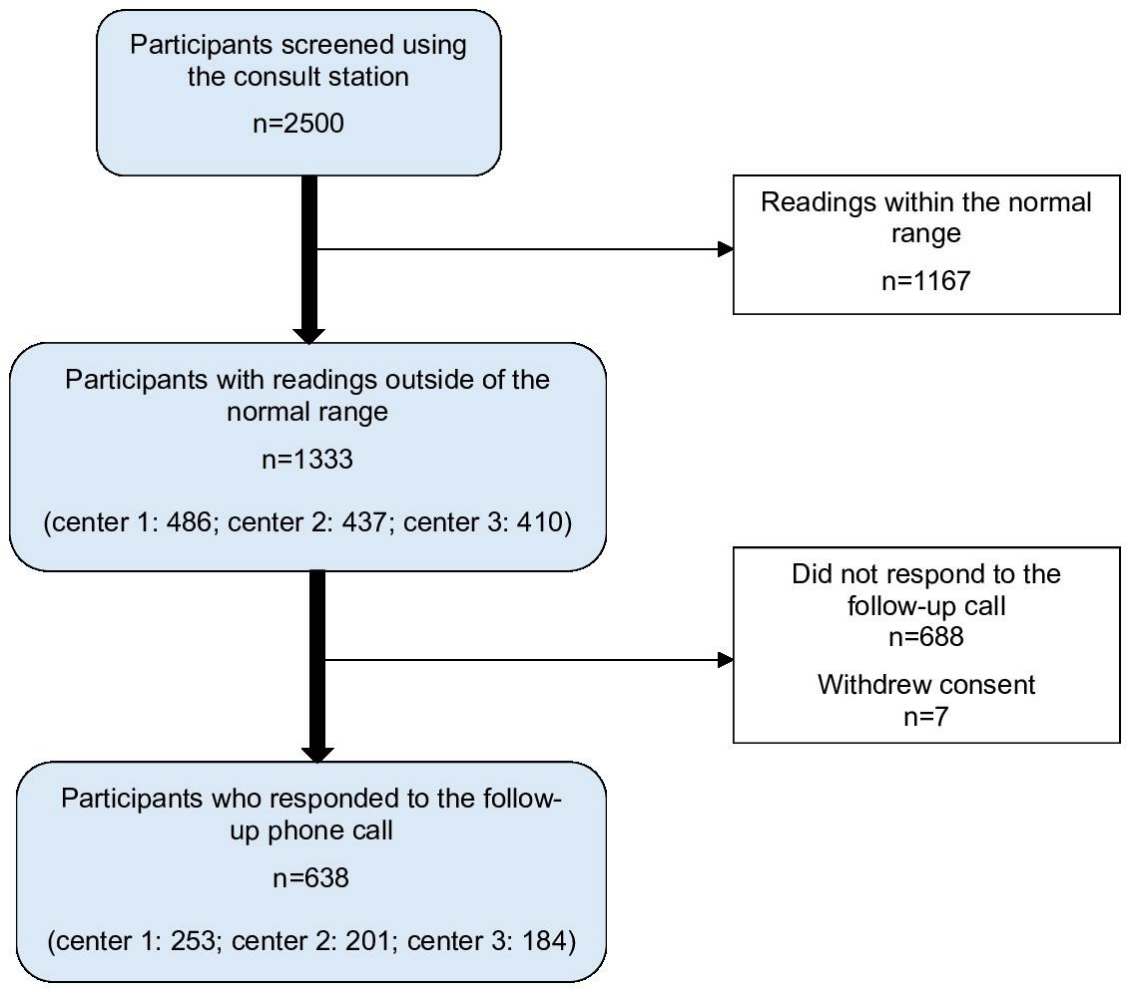
Flow chart of the study participants.

Approximately 6000 people attended the three vaccination centers between July 2021 and September 2021 and were given the opportunity to take part in the study. There were around 2500 people who had a health check using the consult station (2500/6000; 41.7%). In total, 1333 participants (53.3%; 1333/2500) were found to have readings outside the normal range and so were advised to consult a doctor (center 1: N=486; center 2: N=437; center 3: N=410). These participants (N=1333) all provided their contact details, but 7 withdrew their consent and only 638 (47.9%) responded to the follow-up telephone call (center 1: N=253; center 2: N=201; center 3: N=184; see Figure 1).

### Screening Results

**Figure 2.**
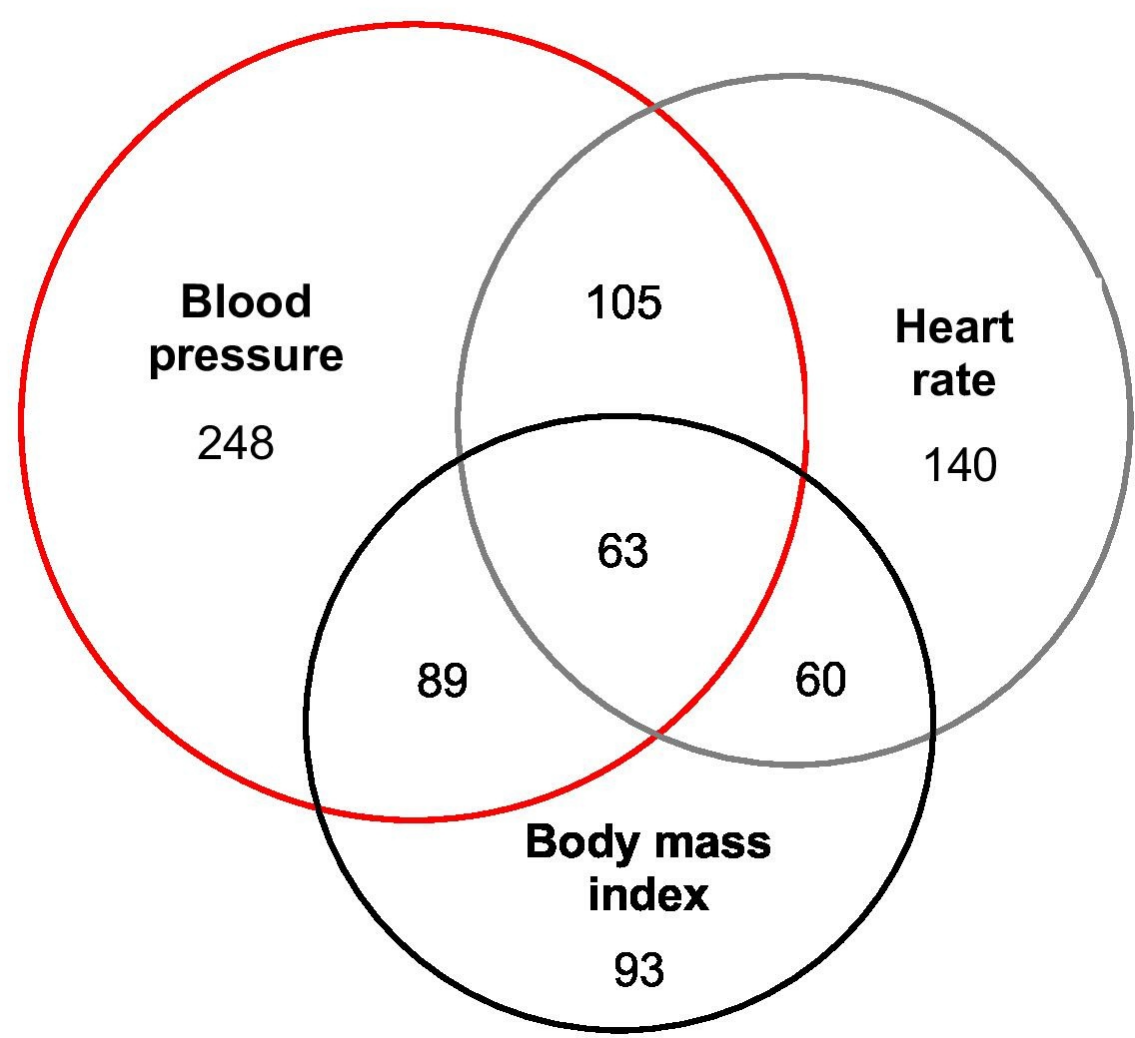
Venn diagram showing the overlap between abnormal readings for body mass index, blood pressure, and heart rate. Note that the abnormal readings for blood pressure include both abnormal diastolic blood pressure and abnormal systolic blood pressure. Participants who had abnormal readings for both of these were counted once.

Results sheets were available for just 1087 of the participants who used the consult station (1087/2500; 43.5%; all at center 1 and center 2). These data were analyzed, and it was found that over a quarter had heart rate, BMI, and blood pressure outside of the normal range (Table 1). The overlap between these abnormal readings is shown as a Venn diagram (Figure 2).

**Table 1.** Consult station screening results based on the available results sheets (total: 1087).

Variable	Statistical values	Number of readings outside normal range, n/N (%)^a^
Body mass index (kg/m^2^), median (IQR) range	26 (23‐30) 14‐53	305/1025 (29.8)
Heart rate (beats per minute), median (IQR) range	75 (67-84) 21-132	368/985 (37.4)
Oxygen saturation (%), mean (SD) range	97.5 (1.6) 75‐99	13/986 (1.3)
Systolic blood pressure (mmHg), median (IQR) range	126 (114‐138) 84‐225	283/1016 (27.9)
Diastolic blood pressure (mmHg), median (IQR) range	80 (73‐89) 42‐136	416/1016 (40.9)
Amsler grid (central visual field)	—^b^	105/1016 (10.3)

a The total number of available readings is lower than the actual number of participants who completed each measure. This is because results sheets were not available for all study participants (eg, none were available from center 3); in addition, certain measures were not obtained for all participants.

b —: not applicable.

### Primary Outcome Measure

Of the 638 participants who responded to the follow-up telephone call, 234 (36.7%) reported that they had consulted a doctor (GP or specialist) since the health check (center 1: 70/253, 27.7% participants; center 2: 53/201, 26.4% participants; center 3: 111/184, 60.3% participants). However, 24.8% of the participants (158/638) reported that they would have consulted a doctor anyway even without the health check (Table 2).

**Table 2.** Responses to the follow-up call questions (n=638).

Question	Responses, n/N (%)
Sex	
Female	394/638 (61.8)
Male	234/638 (36.7)
Missing	10/638 (1.6)
Reason for coming to vaccination center	
Vaccination	447/638 (70.1)
Other reason	189/638 (29.6)
Missing	2/638 (3.1)
Smoker	
Yes	183/638 (28.7)
No	455/638 (71.3)
Registered with GP^a^	
Yes	620/638 (97.2)
No	18/638 (2.8)
Frequency of GP appointments	
<1 per year	212/638 (33.2)
At least 1 per month	302/638 (47.3)
Around 4 per year	104/638 (16.3)
Missing	20/638 (3.1)
Medical history	
Hypertension	122/638 (19.1)
Cardiac arrhythmia	59/638 (9.2)
Pulmonary disease	49/638 (7.7)
Diabetes	50/638 (7.8)
Sleep apnea	42/638 (6.6)
Concomitant diseases^b^	
Yes	253/638 (39.7)
Cardiovascular system	118/638 (18.5)
Nervous system	65/638 (10.2)
Metabolic disorders	56/638 (8.8)
Respiratory system	45/638 (7.1)
Other	129^c^
GP appointment since health check	
Yes	178/638 (27.9)
No	457/638 (71.6)
Missing	3/638 (0.5)
Saw own GP	141/178 (79.2)^d^
Saw another GP	36/178 (20.2)^d^
Missing	1/178 (0.6)^d^
GP confirmed cardiovascular risk factors	59/178 (33.1)^d^
GP did not confirm risk factors	116/178 (65.2)^d^
Missing	3/178 (1.7)^d^
GP initiated regular follow up	56/59 (94.9)^e^
GP did not initiate regular follow up	2/59 (3.4)^e^
Missing	1/59 (1.7)^e^
Seen specialist since health check	
Yes	151/638 (23.7)
No	483/638 (75.7)
Missing	4/638 (0.6)
Seen dietitian since health check	
Yes	92/638 (14.4)
No	537/638 (84.2)
Missing	9/638 (1.4)
Would have seen doctor without health check	
Yes	158/638 (24.8)
No	474/638 (74.3)
Missing	6/638 (0.9)

a GP: general practitioner.

b Note that some patients may have had more than one concomitant disease.

c The percentage is not shown as the number includes participants who are counted more than once because they take more than one drug.

d The percentage only includes participants who saw a GP.

e The percentage only includes participants who saw a GP and had the risk factors confirmed.

Chi-square tests were run to identify potential factors that might predict whether participants consulted a doctor following the health check. The factors that had a *P* value above .2 were the participants’ sex (*P*=.16), their reason for coming to the vaccination center *(P*<.0001), whether they smoked *(P*=.03), whether they were registered with a GP *(P*=.19), and whether they had a history of certain diseases (hypertension, cardiac arrhythmia, pulmonary disease, diabetes, and sleep apnea*; P*<.0001). A multivariate analysis was then run, which included these factors. The results showed that the reason for coming to the vaccination center and a history of certain diseases were statistically significant at the *P*<.05 level *(P*<.001 for both factors). The pattern of results indicated that participants were more likely to see a doctor following the health check if they had come to the center to be vaccinated or if they had a history of certain diseases.

### Secondary Outcome Measures: Differences Between Participants Registered With a General Practitioner and Those Not Registered With a General Practitioner

There were 18 participants (18/638; 2.8%) who reported that they were not registered with a GP. Five of these (27.8%) consulted a doctor following the health check. Due to the lack of data, we did not carry out further analyses.

### Additional Analyses

Additional analyses were run to determine whether there were significant differences between the 3 vaccination centers in terms of the follow-up call results (Table 3). We found that there was a significant difference between the centers in terms of the proportion of participants who were registered with a GP *(P*=.04). Specifically, fewer were registered with a GP at center 2 (94.5%) than at center 3 (98.4%) and center 1 (98.4%). This can be attributed to the relative lack of GPs in this region (101 per 100,000 individuals compared with 129 and 159 per 100,000 population for the other 2 regions) [25]. We also found a significant difference concerning the proportion of participants who consulted a GP following the health check *(P*<.001). This was higher at center 2 (45%) than at the 2 other centers (20.9% and 19.2%). Similarly, a higher proportion of participants at center 2 (34.3%) consulted a medical specialist than at the 2 other centers (17.7% and 20.7*%; P*<.0001). These results indicate that the screening led more participants at center 2 to consult a doctor. However, a higher proportion of participants at this center reported that they would have seen a doctor anyway (50.3% vs 14.5% and 12.0*%; P*<.0001). Of note, the participants at the different centers did not differ significantly in terms of how often they usually saw a doctor *(P*=.82).

**Table 3.** Differences between the vaccination centers.

Question	Center 1, n/N (%)	Center 2, n/N (%)	Center 3, n/N (%)	*P* value (test)
Sex				.43^a^
Female	152/253 (60.1)	120/201 (59.7)	122/184 (66.3)	
Male	97/253 (38.3)	76/201 (37.8)	61/184 (33.2)	
Missing	4/253 (1.6)	5/201 (2.5)	1/184 (0.5)	
Smoker				.02^a^
Yes	87/253 (34.4)	56/201 (27.9)	40/184 (21.7)	
No	166/253 (65.6)	145/201 (72.1)	144/184 (78.3)	
Missing	0/253 (0)	0/201 (0)	0/184 (0)	
Registered with GP^b^				.04^c^
Yes	249/253 (98.4)	190/201 (94.5)	181/184 (98.4)	
No	4/253 (1.6)	11/201 (5.5)	3/184 (1.6)	
Missing	0/253 (0)	0/201 (0)	0/184 (0)	
See GP				.82^e^
<1 per year	90/249^d^ (36.1)	56/188^d^ (29.8)	66/181^d^ (36.5)	
~4 per year	49/249^d^ (19.7)	25/188^d^ (13.3)	30/181^d^ (16.6)	
1 per month+	110/249^d^ (44.2)	107/188^d^ (56.9)	85/181^d^ (47)	
Missing	0/249^d^ (0)	2/188^d^ (1.1)	0/181^d^ (0)	
Seen GP since health check				<.0001^a^
Yes	53/253 (20.9)	90/201 (44.8)	35/184 (19.0)	
No	200/253 (79.1)	110/201 (54.7)	147/184 (79.9)	
(Missing)	0/253 (0)	1/201 (0.5)	2/184 (1.1)	
Saw own GP	39/53^f^ (73.6)	81/90^f^ (90)	21/35^f^ (60)	.001^a^
Saw different GP	13/53^f^ (24.5)	9/90^f^ (10)	14/35^f^ (40)	
(Missing)	1/53^f^ (1.9)	0/90^f^ (0)	0/35^f^ (0)	
GP confirmed cardiovascular risk factors	28/53^f^ (52.8)	15/90^f^ (16.7)	16/35^f^ (45.7)	<.0001^a^
GP did not confirm risk factors	23/53^f^ (43.4)	75/90^f^ (83.3)	18/35^f^ (51.4)	
(Missing)	2/53^f^ (3.8)	0/90^f^ (0)	1/35^f^ (2.9)	
GP initiated regular follow up	27/28^g^ (96.4)	13/15^g^ (86.7)	16/16^g^ (100)	.06^c^
GP did not initiate follow up	0/28^g^ (0)	2/15^g^ (13.3)	0/16^g^ (0)	
(Missing)	1/28^g^ (3.6)	0/15^g^ (0)	0/16^g^ (0)	
Seen specialist since health check				<.0001^a^
Yes	44/253 (17.4)	69/201 (34.3)	38/184 (20.7)	
No	205/253 (81)	132/201 (65.7)	146/184 (79.3)	
Missing	4/253 (1.6)	0/201 (0)	0/184 (0)	
Would have consulted physician without health check				<.0001^a^
Yes	36/253 (14.2)	100/201 (49.8)	22/184 (12.0)	
No	213/253 (84.2)	99/201 (49.3)	162/184 (88)	
Missing	4/253 (1.6)	2/201 (1.0)	0/184 (0)	

a Chi-square test.

b GP: general practitioner.

c Fisher exact test.

d The total only includes participants who were registered with a GP.

e Kuskal-Wallis test.

f The total only includes participants who saw a GP since the health check.

g The total only includes participants who saw a GP and had the cardiovascular risk factors confirmed.

## Discussion

### Principal Findings

In this study, large numbers of people were screened for CVD risk factors during the COVID-19 pandemic using the consult station telemedicine booth. According to the available results sheets, over half had readings outside the normal range, which were mostly for blood pressure (diastolic: 41%; systolic: 28%), heart rate (37%), and BMI (30%). Even though relatively few people reported that they consulted a doctor because of their results (around 12% of the follow-up call respondents), the screening would nevertheless have raised people’s awareness of their risk factors and may have led to beneficial lifestyle changes.

Our study showed that the telemedicine booth was generally well-accepted by the French public, with around 42% of visitors to the vaccination centers choosing to use the device. Although the booth’s enclosed environment may have led to fears of infection during the pandemic, we found that this did not prevent almost half of the visitors from using the consult station and benefiting from the free health check. We speculate that even more people would use the device at other times when there is less fear of infection. Indeed, the privacy provided by the booth is likely to encourage more people to use the device as this could improve user experience.

This high level of acceptance supports the use of telemedicine booths for CVD screening not only during pandemics but also at other times. They could potentially be used to screen large numbers of people, and they have the advantage of low running costs. They would be of particular benefit to regions that lack medical professionals, the so-called “medical deserts” of France, to improve screening services in these regions [13]; the booths could also potentially be used to provide teleconsultations with medical professionals in these regions. The telemedicine booths should ideally be placed in carefully selected locations to target groups of people who are known to have a higher risk of CVD, such as older adults. This has already been done in the United States, where blood pressure checks have been run in Black-owned barbershops to target Black American men who have a high prevalence of hypertension [26].

One of the strengths of our study is that we evaluated the impact of the screening by examining whether people with abnormal results went on to consult a physician. This is an important consideration, as it has been pointed out that people with hypertension may not always seek medical treatment [27]. In our study, only around 12% of the respondents reported that they consulted a doctor because of the screening. This raises the question as to why this was the case, especially as the participants had been explicitly advised to see a doctor. One possibility relates to the fact that the study took place during the COVID-19 pandemic, and so participants may have avoided medical consultations when there was a risk of infection as well as an overwhelmed health system. Another possibility is that some participants may already have been aware of their condition and so they may already have consulted a doctor about it. It is also possible that some participants did not fully understand the dangers of their abnormal readings or the fact that effective treatment options are available from their GP; this consideration is important, as it could potentially be remedied by providing educational material.

Although relatively few participants with abnormal readings consulted a doctor, improvements may nevertheless have occurred. Specifically, it is possible that the participants may have adopted lifestyle changes to improve their health. For instance, they may have searched the internet for advice on how to lose weight or lower their blood pressure. In support of this, a previous study found that many people with obesity prefer to find out about weight management from the internet, friends, and smartphone apps rather than from their health care provider [28].

Our statistical analyses revealed 2 factors that could predict whether participants with abnormal readings would consult a doctor. The first was having a history of certain diseases, such as diabetes. This could be attributed to the fact that patients with these diseases often have regular follow-up appointments with a GP. In addition, they may also be more aware of their need for medical care. The second factor was coming to the center to be vaccinated (as opposed to accompanying someone or for work); this may be because these participants were generally inclined to seek medical care (eg, vaccination) and so they would be more likely to consult a doctor following the screening.

One of the aims of our study was to examine differences between participants who were registered with a GP and those who were not. In France, it has been reported that a substantial proportion of the population (11%) does not have a GP [13]. However, we only identified 18 participants (2.8%) who were not registered with a GP, and so we did not carry out statistical analyses. This low number may be because the people who came to the vaccination centers were generally inclined to seek medical care, including vaccination, and so would also be more likely to register with a GP. We noted that most of the participants who were not registered with a GP were at center 2, which can be attributed to the relative lack of GPs in this region (101 per 100,000 population for the center 2 as opposed to 129 and 159 per 100,000 population for the 2 other centers) [25]. However, this did not appear to affect the postscreening medical consultations, as the participants at this center were also more likely to see a physician following the health check. This latter finding may relate to the fact that over half of the participants at center 2 reported seeing a GP at least once a month, unlike at the other 2 centers; these participants may therefore have been more likely to consult a physician following the health check simply because they were already frequent health care users.

The limitations of this study include the lack of a control group and missing data (no screening results for over half of the participants). In addition, we were unable to link the follow-up results to the initial screening data (except for 55 participants). In addition, it is possible that certain screening measures (eg, blood pressure) may have been affected by the vaccination (eg, prevaccination anxiety or postvaccination side effects) [29]. The results might also have been affected by bias in terms of the study participants, as those who took part were at a vaccination center and so were presumably inclined to seek medical care. Finally, we did not assess whether the screening had an impact on risk factor control over the long term.

In the future, it would be helpful to run further studies to identify the most effective locations for screening, with the aim of targeting people who rarely consult a GP. In addition, it would be helpful to develop educational pamphlets or videos that explain CVD risk factors and the treatment options that are available, as this could potentially increase the number of people who seek medical care. Additional screening measures could also be introduced to detect other diseases, such as the ankle-brachial index, which can indicate whether someone has peripheral artery disease, a prevalent but currently under-diagnosed condition [30]. It would also be beneficial to introduce mHealth systems, such as mobile phone-based lifestyle interventions, for patients who are found to have CVD risk factors. These has previously been shown to effectively lower the risk of atherosclerotic CVD and increase peak oxygen consumption and so could potentially greatly increase the health benefits following screening [31,32].

As a final note, there are current plans for the consult station to be used for screening in regions of France where there is a high prevalence of hypertension and a lack of medical services. Our medical group has also started sending mobile health buses to medically underserved towns in the Hauts de France region, which aim to promote healthy lifestyles and prevent disease through patient education.

### Conclusions

By placing the consult station in vaccination centers during the COVID-19 pandemic, it was possible to screen large numbers of people for CVD risk factors. Our results support the use of telemedicine booths for health screening, particularly in regions that lack medical professionals. In the future, it would be helpful to develop educational material to ensure that users understand the significance of any abnormal results and the treatment options that are available.

## Abbreviations

bpm: beats per minute
CVD: cardiovascular disease
GP: general practitioner
